# Novel Approach to the Treatment of Neuropathic Pain Using a Combination with Palmitoylethanolamide and *Equisetum arvense* L. in an In Vitro Study

**DOI:** 10.3390/ijms24065503

**Published:** 2023-03-13

**Authors:** Sara Ruga, Rebecca Galla, Sara Ferrari, Marco Invernizzi, Francesca Uberti

**Affiliations:** 1 Laboratory of Physiology, Department of Translational Medicine, University of Piemonte Orientale, Via Solaroli 17, 28100 Novara, Italy; 2 Noivita Srls, Spin-Off, Via Alfieri 3, 28100 Novara, Italy; 3 Physical and Rehabilitation Medicine, Department of Health Sciences, University of Eastern Piedmont, 28100 Novara, Italy; 4 Translational Medicine, Dipartimento Attività Integrate Ricerca e Innovazione (DAIRI), Azienda Ospedaliera SS, Antonio e Biagio e Cesare Arrigo, 15121 Alessandria, Italy

**Keywords:** neuropathic pain, peripheral neuraxis damage, palmitoylethanolamide, *Equisetum arvense* L., intestinal adsorption, Schwann cells

## Abstract

Neuropathic pain is a typical patient disorder resulting from damage and dysfunction of the peripheral neuraxis. Injury to peripheral nerves in the upper extremities can result in a lifelong reduction in quality of life and a devastating loss of sensory and motor function. Since some standard pharmaceutical therapies can cause dependence or intolerance, nonpharmacological treatments have gained great interest in recent years. In this context, the beneficial effects of a new combination of palmitoylethanolamide and *Equisetum arvense* L. are evaluated in the present study. The bioavailability of the combination was initially analyzed in a 3D intestinal barrier simulating oral intake to analyze its absorption/biodistribution and exclude cytotoxicity. In a further step, a 3D nerve tissue model was performed to study the biological effects of the combination during the key mechanisms leading to peripheral neuropathy. Our results demonstrate that the combination successfully crossed the intestinal barrier and reached the target site, modulating the nerve recovery mechanism after Schwann cell injury and offering the initial response of relieving pain. This work supported the efficacy of palmitoylethanolamide and *Equisetum arvense* L. in reducing neuropathy and modifying the major pain mechanisms, outlining a possible alternative nutraceutical approach.

## 1. Introduction

Recently, the International Association for the Study of Pain (IASP) released a definition of neuropathic pain (NP) in order to clarify the many existing definitions. It has therefore been defined as “pain caused by an injury or disease of the somatosensory nervous system” [1]. Normally, the somatosensory system allows the perception of touch, pressure, pain, temperature, position, movement, and vibration. Injury or disease can damage peripheral or central nerve fibers by interrupting or altering the pain signals that nerves send and receive from all over the body [2]. Indeed, NP is caused by chronic lesions or pathologies within the somatosensory nervous system with damage and loss of function of nerve cells [3].

Furthermore, a factor that interferes with the development of a therapeutic strategy for NP is the difficulty in estimating its incidence and prevalence due to the lack of simple diagnostic criteria that make broad epidemiological investigations possible in the general population [4]. Thus, not all patients with peripheral neuropathy or a central nervous system injury develop NP; however, the prevalence of NP increased to 60% in those with severe clinical neuropathy [5]. To date, NP has become a massive burden in recent years, affecting approximately 7–8% of the global population [6] and associating very high annual healthcare costs for the treatment, care, and rehabilitation with a cost of approximately €2.2 billion/year in Europe and $150 billion/year in the United States [7]. Therefore, injuries to peripheral nerves affect over one million people every year worldwide and may cause significant lifelong disability, with serious impacts (both physical and psychological) on the patients’ lives [8]. Pain research has focused on understanding plastic changes in the nervous system after nerve injury, identifying novel therapeutic targets, and facilitating the transfer of knowledge from cell models to clinical practice [5,9]. Therefore, from a pathophysiologic point of view, neuronal hyperactivity and/or hyperexcitability, which occur after nerve damage, are the primary causes of persistent pain [3]. Among the theories explaining the mechanisms related to central neuropathy, it is also important to highlight that intracellular signaling, microglial activation, neurotrophic factors, and increased levels of pro-inflammatory and inflammatory mediators, as well as oxidative stress (OS), are involved in abnormal pain perception [6].

Recent data from experiments conducted on chronic constriction-induced neuropathic pain in the rat sciatic nerve showed that radical oxygen species (ROS)-induced OS downregulates the level of Sirtuin 3 (SIRT3), a key player in several biological processes, including mitochondrial functions [10]. Furthermore, the particular environment created by OS stimulates inflammatory cells such as lymphocytes, monocytes, neutrophils, basophils, and eosinophils, causing them to release a variety of chemicals such as proinflammatory cytokines, vasoactive amines, peptides, and eicosanoids. Indeed, upregulation of proinflammatory mediators such as TNF-α and IL-1β has been shown to be responsible for neuropathic pain [6]. Likewise, another important player in the development of NP is gamma-aminobutyric acid (GABA) [9]. It is worth noting that GABA is a potent inhibitory neurotransmitter, and activation of both its GABAA and GABAB receptors reduces both neuronal hyperexcitability and nociception in the neuropathic model [11]. Therefore, pain modulation is a top-down process: changes in GABAergic function are assumed to affect synaptic plasticity in related proteins or genes. This synaptic plasticity causes decreased GABA levels, decreased expression of the L-glutamate decarboxylation enzyme, which is catalyzed by the glutamic acid decarboxylase (GAD) enzyme, GABA transporters, and GABA receptor dysfunction in neuropathic pain [12,13]. In this regard, hyperexcitability can also be modulated by the endocannabinoid system, which acts as a regulator of synaptic transmission [14]. Clinical evidence has been reported for the role of the endocannabinoid system in pain management, as an imbalance in the physiological signaling of the endocannabinoid system can induce symptoms typical of chronic neuropathic pain [9,14]. The endocannabinoid system factors are highly susceptible to molecular alterations, and such events are observed in chronic NP pathogenesis [9,15]. Indeed, it has been reported that the expression of cannabinoid receptors 1 and 2 (CB1R and CB2R), the endocannabinoid system synthesis machinery, and the expression of fatty acid amide hydrolase (FAAH) are improved in chronic NP. Consequently, there is an elevation in endocannabinoid system synthesis due to the activation of FAAH and an inhibition of alternative catabolic pathways involving cytochrome p450 (CYP), cyclooxygenase-2 (COX-2), and lipoxygenases. This scenario leads to the generation of inflammatory mediators, such as prostaglandins, which influence neuron excitability and modulate CB1R-mediated negative feedback in excitatory synapses, contributing to the hyperalgesia mechanism [9,16].

The fact that the OS, GABA, and endocannabinoid systems are involved in the pathophysiological state of pain makes these systems valid targets for chronic NP treatment. Nowadays, treatments for chronic NP consist of different lines of therapy that are chosen according to the patient’s condition [17]. Therefore, available strategies to improve axonal regeneration, neuronal survival, myelination, and reinnervation of target organs after nerve injury include surgical and non-surgical therapeutic approaches [18]. Despite the progress in understanding the pathophysiology of peripheral nerve injuries, the advancement in reconstructive microsurgery, and the innovations in the fields of tissue engineering and regenerative medicine, there are no repair techniques or therapeutic approaches that can ensure full recovery of normal sensorimotor functions in adult patients following severe nerve injuries [7]. Therefore, alternative and potentially more efficacious treatments to ameliorate the regeneration of damaged peripheral nerves should be sought, but also supportive accompanying strategies are worth more consideration. Although there is widespread availability of targeted therapies for PN, the low increases in responses compared to placebo have led to the failure of many new drugs [3,7,17,19,20]. In this context, plant-derived compounds have been suggested to have potential beneficial effects on peripheral nerve regeneration [18], since they are defined as "nutraceuticals" (dietary supplements and herbal/natural products) due to their low cost, high nutritional and therapeutic values, and ability to bind to multiple molecular targets with a pharmacologically beneficial effect on health [21]. In this context, recent research has highlighted the anti-inflammatory and immune-modulating roles of palmitoylethanolamide (PEA), as it has a neuroprotective effect, acting on several molecular targets in the central and peripheral nervous systems (PNS) [22,23]. In addition, PEA is an endogenous agonist of the endocannabinoid system, acting on CB1 and CB2 receptors, and has been described as a valuable option to successfully modulate chronic NP in animal models, providing an alternative treatment for patients that do not respond well to other pharmacological therapies [16,24,25]. As a consequence, CB1 expression is induced by neurotrophins, such as brain-derived neurotrophic factor (BDNF) and nerve growth factor (NGF), which allow proper nerve transmission by regulating the pain sensation [26,27]. However, although this molecule shows remarkable properties, it also shows some limitations that affect its use orally. In particular, some negative aspects of PEA are its highly lipophilic nature and the large particle size of its native state. Furthermore, it has a low rate of oral absorption in aqueous vehicles, so its dissolution rate is often the limiting step for its poor oral bioavailability [28]. Furthermore, according to some reports, PEA reaches its maximum plasma concentration in 2 h, so its levels quickly return to normal, indicating that the effect is only temporary [29]. In view of these tests, PEA is generally used in micronized form, as it allows smaller particles, providing a larger surface area, to be easily absorbed [30]. Indeed, micronized or ultra-micronized formulations of PEA have been shown to maximize the bioavailability and efficacy of the compound [27]. Moreover, these limitations can be easily overcome with new technologies or through combined action with other molecules that, on the one hand, facilitate its absorption [23,31] and, on the other hand, enhance its anti-inflammatory and protective action in a tissue-specific manner [23]. Among the various antioxidant molecules capable of performing this function, *Equisetum arvense* L. (*Equisetum A.*L.) has attracted particular attention in pain management [32]. This herbal medicine, commonly known as horsetail, is a rich source of phenolic compounds, flavonoids, and phenolic acids with antioxidant capacity [33,34]. Numerous studies have described distinct biological effects of *Equisetum A.*L. extracts, for instance their antioxidant and neuroprotective properties [35], since they play an important role in the oxidative stress response mechanism and in the activation of SIRT1, which mediates chronic pain associated with peripheral nerve injury [32,36]. Indeed, recent scientific evidence revealed that SIRT1 agonists can relieve chronic pain through regulating inflammation, oxidative stress, synaptic plasticity, and mitochondrial dysfunction; thus, activating SIRT1 becomes a promising treatment for chronic pain [36]. Based on this scientific evidence, several studies have demonstrated nutraceuticals superior performances when co-delivered, and, therefore, the concept of synergism has been established for various bioactives. Generally, the synergistic effects of nutraceutical compounds can prevent or treat chronic diseases with better therapeutic effects and efficacy while ensuring that toxicity effects are under control [37]. Therefore, in our specific case, the synergistic effects of PEA and *Equisetum A.*L. could improve significant changes in the biological effects and/or the bioavailability of components.

Based on the anti-inflammatory and analgesic capacity of PEA and the antioxidant properties of *Equisetum A.*L., it was hypothesized to combine these two extracts to obtain a new dietary supplement able to modulate specific key mechanisms of NP. Therefore, it is possible to state that the use of dietary supplements becomes of fundamental importance in hypothesizing a new and innovative therapeutic approach with the goal of improving the quality of life of NP patients. In this context, the aim of the current study is to evaluate a new food supplement based on PEA combined with *Equisetum A.*L. in order to modulate the mechanism that leads to peripheral nerve injury, which then flows into NP.

## 2. Results

### 2.1. Safety Analysis of Different Concentrations of PEA and Equisetum on CaCo-2 Cells

Several experiments were performed on the CaCo-2 cell line to exclude any cytotoxic effect. In particular, cell viability and ROS production were investigated at 3 h and 6 h, the maximum and minimum times of PEA absorption [23]. For this reason, the analysis was carried out by testing PEA 80mesh and *Equisetum A.*L. (in a range of 0.1 µM to 0.4 µM and from 25 µg/mL to 100 µg/mL, respectively) and comparing them with another PEA form present in the market, named ultra-micronized PEA (PEA-um). As demonstrated in Table 1, both PEA 80mesh and PEA-um improved cell viability in all tested concentrations, excluding any cytotoxic effect (*p* < 0.05). It should be emphasized that cells treated with PEA 80mesh demonstrated a highly beneficial effect, improving cell viability better than the other PEA form (*p* < 0.05). Considering only PEA 80mesh, as shown in Table 1, the concentration of 0.2 µM showed a more significant improvement in cell viability compared to the other concentrations tested (about 8% compared to 0.1 µM and about 24% compared to 0.4 µM, *p <* 0.05), suggesting that this treatment dosage may support improved bowel activity. At the same time, the same test was performed by also analyzing the beneficial effect of *Equisetum A.*L., and, as shown in Table 1, all the concentrations tested were able to improve cell viability (*p* < 0.05), confirming, also in this case, the absence of a toxic effect. More in detail, at 3 h of stimulation, the three concentrations tested revealed a similar trend, suggesting that there was no biological difference between them. However, at 6 h *Equisetum A.*L., 50 µg/ml was able to maintain higher cell viability than the other concentrations tested (about 32% compared to 25 µg/ml and about 9% compared to 100 µg/ml, *p* < 0.05), suggesting a possible long-term effect on mitochondrial metabolism at the intestinal level. Consequently, the ROS production was analyzed on CaCo-2 cells to confirm the safety of PEA 80mesh and *Equisetum A.*L., analyzing the effect at the same time previously explored. As shown in Table 2, none of the concentrations tested increased ROS production. In particular, PEA 80mesh 0.2 µM was able to maintain low ROS levels throughout the stimulation period better than PEAum, and *Equisetum A.*L. 50 µg/ml demonstrated antioxidant capacity by reducing the quantity of ROS produced compared to the control (*p* < 0.05).

For this reason, the concentrations chosen for the final formulation were PEA 80mesh 0.2 µM, and *Equisetum A.*L. 50 µg/mL (named EquiPEA™). Indeed, when these agents were added together, the beneficial effects on cell viability and ROS production were amplified compared to single agents (*p* < 0.05), confirming the absence of toxic events and the safety of the combination.

### 2.2. Permeability and Absorption Mechanisms Analyzed in an In Vitro Intestinal Barrier Model

**Figure 1:**
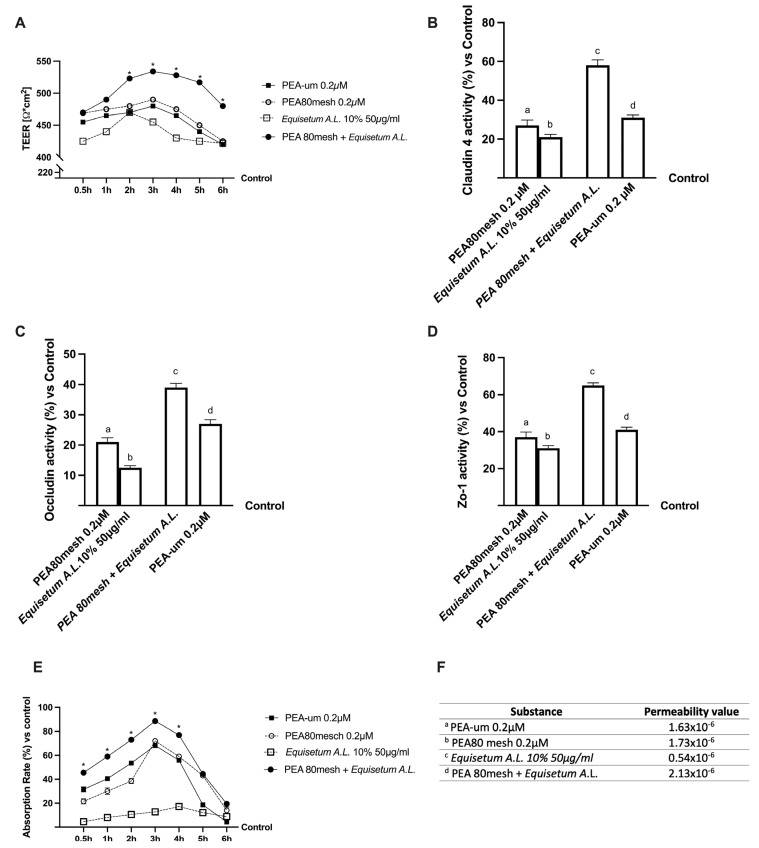
Permeability study on CaCo-2 cells. In (**A**) TEER Value using EVOM3; from (**B**–**D**) the analysis of TJ measured by ELISA test (Claudin1, Occludin and Human Tight Junction Protein 1 (Zo-1), respectively); in (**E**) evaluation of passage through the intestinal barrier by fluorescent tracer; in (**F**): the Papp values in which data < 0.2 × 10^−6^ cm/s means very poor absorption with a bioavailability < 1%, data between 0.2 × 10^−6^ and 2 × 10^−6^ cm/s with bioavailability between 1 and 90%, and data > 2 × 10^−6^ cm/s means very good absorption with a bioavailability over 90%. *Equisetum A.*L. = *Equisetum arvense* L.; PEA-um = PEA ultra-micronized. From (**B**–**E**) data are the means ± SD (%) of five independent experiments performed in triplicate and normalized to control values (the 0% line). PEA-um = ultra-micronized PEA. In (**A**): * *p* < 0.05 vs. PEA80mesh 0.2 µM. In (**B**): a–d *p* < 0.05 vs. control; a *p* < 0.05 vs. b, c; b *p* < 0.05 vs. c, d; c *p* < 0.05 vs. d. In (**C**): a–d *p* < 0.05 vs. control; a *p* < 0.05 vs. b–d; b *p* < 0.05 vs. c, d; c *p* < 0.05 vs. d. In (**D**): a–d *p* < 0.05 vs. control; a *p* < 0.05 vs. b, c; b *p* < 0.05 vs. c, d; c *p* < 0.05 vs. d. In (**E**): * *p* < 0.05 vs. PEA80mesh 0.2 µM. In (**F**): a *p* < 0.05 vs. c, d; b *p* < 0.05 vs. c, d; c *p* < 0.05 vs. d.

Further experiments were conducted to obtain important information about the intestinal absorption and the transport mechanism of PEA 80mesh combined with *Equisetum A.*L. compared to PEA-um, using an in vitro intestinal barrier able to mimic the human physiology of the small intestine. In particular, the transepithelial electrical resistance (TEER), the values of the apparent permeability coefficient (Papp), and the Tigh Junction activity (TJ) were evaluated. The TEER analysis showed that all PEA forms (Figure 1A) are able to maintain epithelial integrity and the ionic conductance of the paracellular pathway, also confirming the peak in permeability at 3 h. Similarly, *Equisetum A.*L. maintains epithelial integrity but suggests poor bioavailability (*p* < 0.05). The combination was able to improve the absorption rate with a more physiological behavior than the single components and to PEA-um (*p* < 0.05) while maintaining the epithelial integrity and increasing the ion flow of paracellular exchanges through the intestinal epithelium (*p* < 0.05). In order to confirm the correct functioning of the intestinal epithelium, the TJ activity (Figure 1B–D) and the permeability rate (Figure 1E,F) were explored. The data obtained from TJ analysis demonstrated that the combination of PEA 80mesh 0.2 µM and *Equisetum A.*L. 50 µg/ml has a better effect compared to the single agents, confirming the result previously obtained about the chosen concentration. Indeed, Claudin-1, Occludin, and Human Tight Junction Protein 1 (ZO-1) activities were improved by the combination of PEA 80mesh 0.2 µM and *Equisetum A.*L. 50 µg/ml compared to PEA-um (about 47.5%, 31%, and 37%, respectively). Finally, the analysis of the basolateral environment confirmed our previous findings, indicating that the permeability rate of PEA 80mesh 0.2 µM combined with *Equisetum A.*L. 50 µg/ml was higher than PEA-um (*p* < 0.05) and the main effect was evident at 3 h of stimulation (about 29%, *p* < 0.05).

All these results revealed that the combination of PEA 80mesh 0.2 µM and *Equisetum A.*L. 50 µg/ml has a better absorption profile compared to PEA-um, confirming an improvement in the bioavailability and suggesting a synergistic effect of the combination of PEA 80mesh 0.2 µM and *Equisetum A.*L. 50 µg/ml to improve intestinal absorption.

### 2.3. Effect of the Combination of PEA 80Mesh and Equisetum arvense on 3D EngNT Co-Cultures

**Figure 2:**
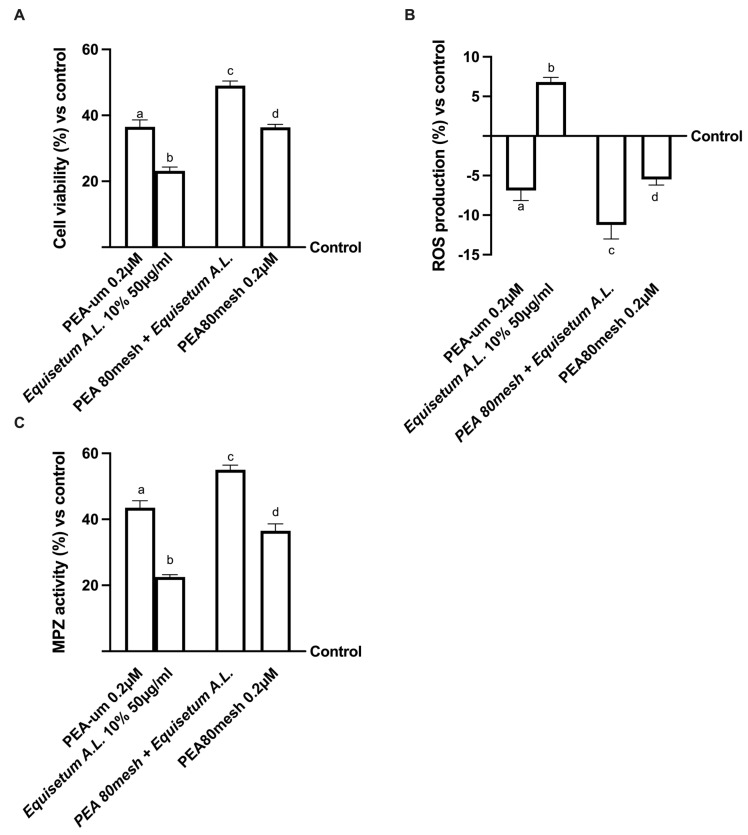
Analysis of EquiPEA™ on 3D EngNT in physiological conditions In (**A**) mitochondrial metabolism measured by the MTT test; (**B**) ROS production by cytochrome C reduction; and (**C**) myelin protein zero (MPZ) measured by the ELISA test. The abbreviations are the same as those reported in Figure 1. The data are the mean ± SD of five independent experiments performed in triplicate and normalized to control values (0% line). In (**A**): a–d *p* < 0.05 vs. control; a *p* < 0.05 vs. b–d; b *p* < 0.05 vs. c, d; c *p* < 0.05 vs. d. In (**B**): a–d *p* < 0.05 vs. control; a *p* < 0.05 vs. b–d; b *p* < 0.05 vs. c, d; c *p* < 0.05 vs. d. In (**C**): a–d *p* < 0.05 vs. control; a *p* < 0.05 vs. b–d; b *p* < 0.05 vs. c, d; c *p* < 0.05 vs. d.

Since the treatments with PEA, in all its forms, are considered an effective remedy for treating peripheral nerve damages, further analyses were conducted on 3D engineered neural tissue (3D EngNT) to investigate the ability of PEA 80mesh combined with *Equisetum A.*L. to reach and act on the peripheral nerve after intestinal passage. As shown in Figure 2, all PEA forms tested can reach the target after intestinal passage without any negative effect on mitochondrial metabolism or oxidative stress (*p* < 0.05). In particular, the combination of PEA 80mesh 0.2 µM and *Equisetum A.*L. 50 µg/mL was able to amplify the cell viability (*p* < 0.05, Figure 2A) compared to PEA-um, demonstrating an ability to maintain the mitochondrial well-being and, at the same time, demonstrating an ability to reduce the amount of ROS production (approximately 26% and 83%, respectively, *p* < 0.05, Figure 2B). The data revealed also the synergistic effect of the two substances on physiological production due to *Equisetum A.*L., which remained within the physiological range. This production (7% vs. control) is useful to maintain physiological homeostasis. These positive effects were further confirmed by the analysis of myelin protein zero (MPZ), a structural protein required for normal peripheral nerve myelination (Figure 2C). As expected, both PEA forms improved MPZ activity (*p* < 0.05) compared to the control. However, the presence of *Equisetum A.*L. in PEA 80mesh significantly improved this marker (approximately 27%, *p* < 0.05) compared to PEA-um, confirming the synergistic effect of the substances. Therefore, these data, obtained under physiological conditions, demonstrate that the combination of PEA 80mesh and *Equisetum A.*L. acts at the PNS level and greatly influences myelin sheath protection.

### 2.4. Biological Effects of the Combination PEA 80Mesh Plus Equisetum A.L. on an In Vitro Model of Peripheral Nervous Injury (PNI)

**Figure 3:**
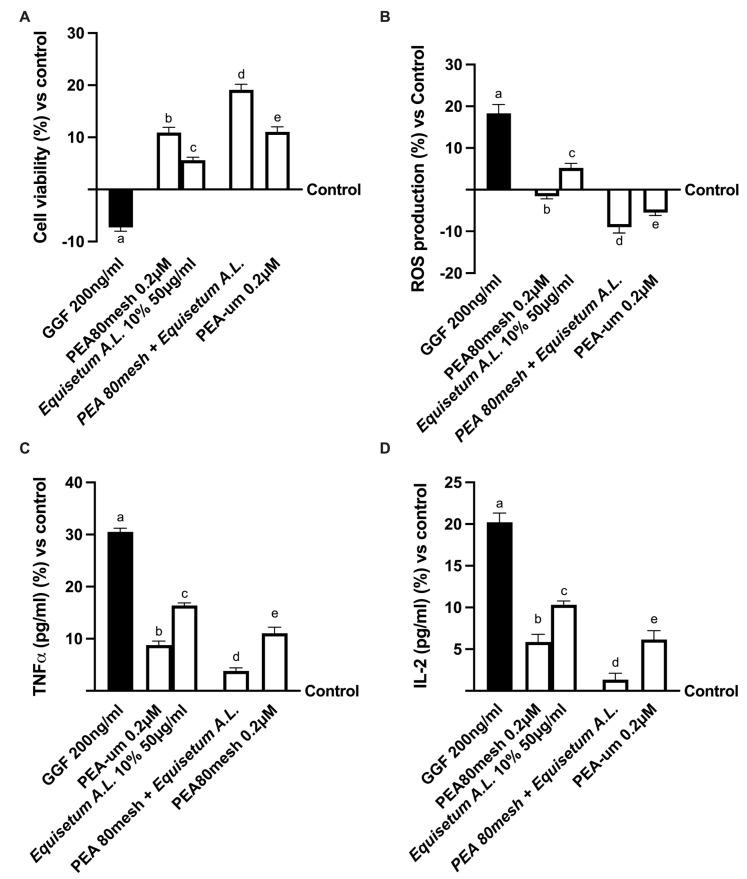
Analysis of PEA 80mesh plus *Equisetum A.*L. effects under PNI conditions In (**A**) mitochondrial metabolism by MTT; in (**B**) nerve growth factor (NGF) determination by ELISA test; in (**C**) TNFa quantification by ELISA test; and in (**D**) IL-1b analysis by ELISA test. The data are the means ± SD (%) of five independent experiments performed in triplicate and normalized to control values (0% line). GGF = glial growth factor 2; the other abbreviations are the same as those reported in Figure 1. In (**A**): a–e *p* < 0.05 vs. control; a *p* < 0.05 vs. b–e; b *p* < 0.05 vs. c, d; c *p* < 0.05 vs. d, e; d *p* < 0.05 vs. e. In (**B**): a, c–e *p* < 0.05 vs. control; a *p* < 0.05 vs. b–e; b *p* < 0.05 vs. c, d; c *p* < 0.05 vs. d, e; d *p* < 0.05 vs. e. In (**C**): a–c, e *p* < 0.05 vs. control; a *p* < 0.05 vs. b–e; b *p* < 0.05 vs. c–e; c *p* < 0.05 vs. d, e; d *p* < 0.05 vs. e. In (**C**): a–c, e *p* < 0.05 vs. control; a *p* < 0.05 vs. b–e; b *p* < 0.05 vs. c, d; c *p* < 0.05 vs. d, e; d *p* < 0.05 vs. e. In (**D**): a–c, e *p* < 0.05 vs. control; a *p* < 0.05 vs. b–e; b *p* < 0.05 vs. c, d; c *p* < 0.05 vs. d, e; d *p* < 0.05 vs. e.

Since the study must mimic the peripheral nerve tissue damage in vitro, the 3D EngNT was pre-treated starting from day 14 of maturation with 200 ng/mL glial growth factor 2 (GGF) to reproduce robust demyelination before stimulation with the same agents used before. In this context, further experiments were conducted by analyzing the effects on mitochondrial metabolism and ROS production (Figure 3A,B). In particular, nerve tissue treated with only 200 ng/mL GGF significantly reduced the biological activity of the nerve and improved ROS production compared to the control (*p* < 0.05). On the contrary, both of these negative conditions were significantly counteracted by the presence of all forms of PEA. In particular, the stimulation with PEA 80mesch combined with *Equisetum A.*L. was able to improve cell viability compared to PEA 80mesh alone (about 90%, *p* < 0.05) and to PEA-um (about 73%, *p* < 0.05). At the same time, it is also able to reduce the oxidative stress produced during the damage better than PEA 80mesh alone (about 80%, *p* < 0.05) and PEA-um (about 71%, *p* < 0.05), supporting the important findings previously observed about the synergic effect of the substances.

In addition, this recovery mechanism was also confirmed by the analyses of the inflammatory markers mainly involved in neuropathy, such as TNFα and IL-2 (Figure 3C,D). Effectively, the beneficial properties of all agents tested could counteract the inflammation process activated by 200 ng/mL GGF (*p* < 0.05) on both markers. Specifically, GGF pre-treatment improved the inflammation response compared to the control (about 30% for TNFα and 20% for IL-2, *p* < 0.05). However, the successive treatment with the combination of PEA 80mesh plus *Equisetum A.*L. was able to reduce the damage better than PEA 80mesh alone (*p* < 0.05) or PEA-um (approximately 57% for TNFα and 78% for IL-2, *p* < 0.05).

**Figure 4:**
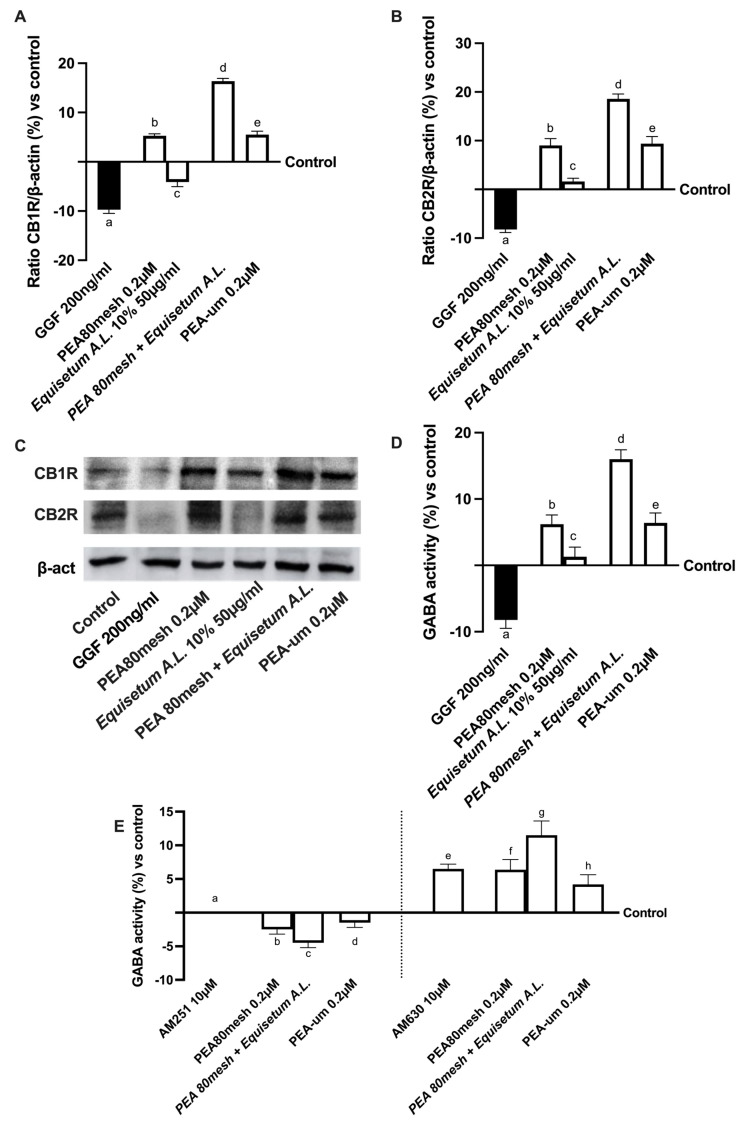
Analysis of EquiPEA™ on CB1/2 receptors and GABA activity under PNI conditions In (**A**) cannabinoid receptor type 1 (CB1R) and in (**B**) cannabinoid receptor type 2 (CB2R) densitometric analysis of the specific Western blot, which is reported as an example in (**C**); in (**D**,**E**) gamma-aminobutyric acid (GABA) activity by ELISA test in the absence or presence of CB1/2 blockers. The data are the mean ± SD (%) of five independent experiments performed in triplicate and normalized to control values (the 0% line). The abbreviations are the same as those reported in Figure 3. In (**A**): a, b, d, e, *p* < 0.05 vs. control; a *p* < 0.05 vs. b–e; b *p* < 0.05 vs. c, d; c *p* < 0.05 vs. d, e; d *p* < 0.05 vs. e. In (**B**): a, b, d, e, *p* < 0.05 vs. control; a *p* < 0.05 vs. b–e; b *p* < 0.05 vs. c, d; c *p* < 0.05 vs. d, e; d *p* < 0.05 vs. e. In (**D**): a, b, d, e *p* < 0.05 vs. control; a *p* < 0.05 vs. b–e; b *p* < 0.05 vs. c, d; c *p* < 0.05 vs. d, e; d *p* < 0.05 vs. e. In (**E**): e–g *p* < 0.05 vs. control; e *p* < 0.05 vs. g; f *p* < 0.05 vs. g; g *p* < 0.05 vs. h.

In addition, since the main target of PEA is the endocannabinoid system, the biological mechanisms of both CB1 and CB2 receptors and their role in inducing GABAergic signaling under PNI conditions were evaluated. Unsurprisingly, as shown in Figure 4 (from A to C), all the considered PEA forms induced both CB1 and CB2 receptor expressions (*p* < 0.05), confirming the crucial role of this bioactive lipid in modulating the endocannabinoid system and changing the behavior due to pain. Also in this case, the use of *Equisetum A.*L. 50 µg/mL in the formulation amplifies the beneficial effect induced by PEA 80mesh (approximately 2 times more for CB1 and one time more for CB2), especially compared to PEA-um (about 2 times more for CB1 and one time more for CB2), suggesting that this formulation acts modulating the endocannabinoid system to reduce the damage. Furthermore, the beneficial effect exerted by PEA was confirmed by the analysis of GABA activity (Figure 4D), in which functionality was statistically increased by all PEA forms compared to control (*p* < 0.05), and thanks to *Equisetum A.*L. 50 µg/mL, PEA 80mesh amplified its effects by 1.5 times, and this effect was also better than PEA-um (*p* < 0.05). Since the basis of the analgesic effect of PEA depends on the relationship between the endocannabinoid system and GABA activation, additional experiments investigate the role of GABA in the presence of CB1 and CB2 blockers (10 µM AM251 and AM60) added 30 min before the addiction of PEA forms and *Equisetum A.*L. (Figure 4E). In particular, it can be observed that the block of CB1 receptor mediated by antagonist AM 251 inhibited the GABA activity, whereas the use of AM630 antagonist of CB2 receptor did not influence the GABA activity, which was maintained at the basal level. The presence of all PEA forms confirmed the GABA activity; indeed, in the presence of CB1 antagonists, the GABAergic system was prevented, and this condition was maintained also during the presence of *Equisetum A.*L. (*p* < 0.05 vs. control), supporting the active role exerted by PEA on CB1 to induce analgesic effect.

Therefore, it is possible to confirm the beneficial role exerted by the combination in modulating the main molecular mechanisms involved in the PNI condition in vitro.

**Figure 5:**
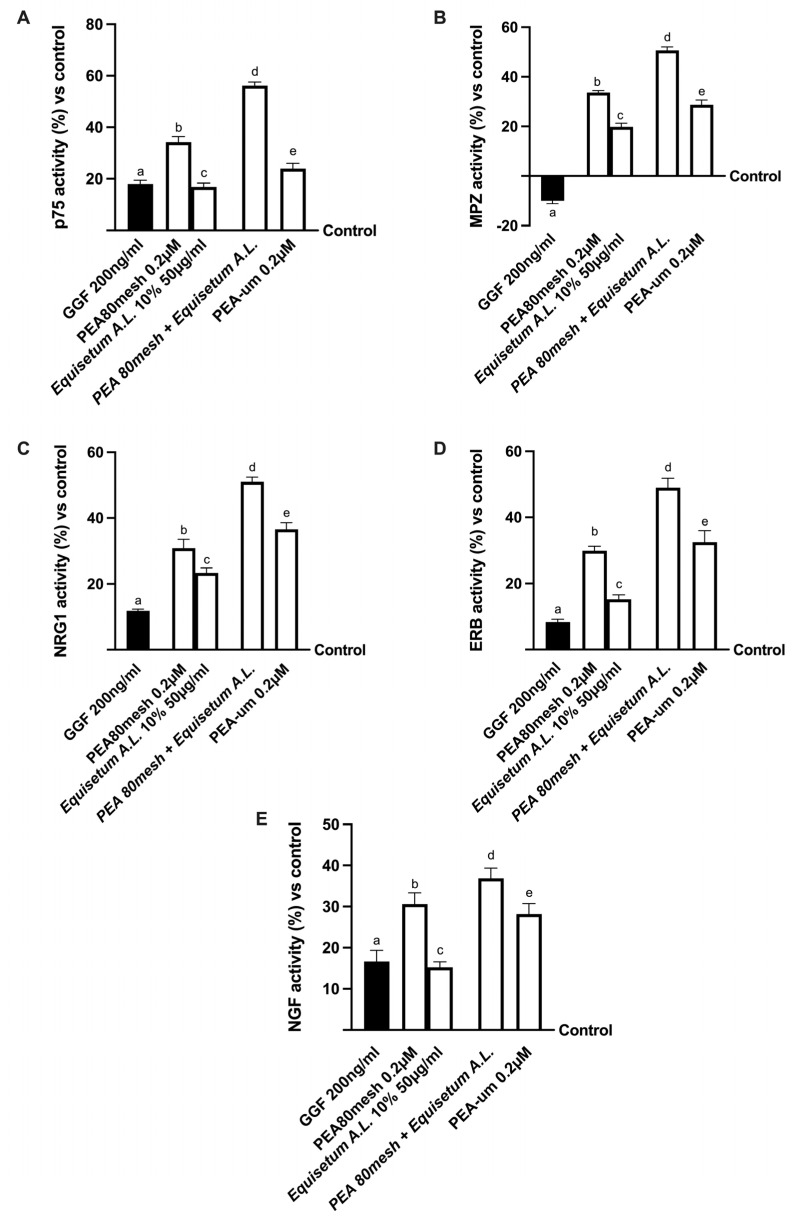
Analysis of Schwann cell activity under PNI conditions In (**A**) p75 activity by ELISA test; in (**B**) myelin protein zero (MPZ) by ELISA test; in (**C**) neuregulin 1 (NRG1) by ELISA test; and in (**D**) estrogen receptor beta (ERb) by ELISA test. The data are the mean ± SD (%) of five independent experiments performed in triplicate and normalized to control values (the 0% line). The abbreviations are the same as those reported in Figure 3. In (**A**): a–e *p* < 0.05 vs. control; a *p* < 0.05 vs. b, d; b *p* < 0.05 vs. c–e; c *p* < 0.05 vs. d; d *p* < 0.05 vs. e. In (**B**): a–e *p* < 0.05 vs. control; a *p* < 0.05 vs. b–e; b *p* < 0.05 vs. c–e; c *p* < 0.05 vs. d, e; d *p* < 0.05 vs. e. In (**C**): a–e *p* < 0.05 vs. control; a *p* < 0.05 vs. b–e; b *p* < 0.05 vs. d; c *p* < 0.05 vs. d, e; d *p* < 0.05 vs. e. In (**D**): a–e *p* < 0.05 vs. control; a *p* < 0.05 vs. b–e; b *p* < 0.05 vs. c, d; c *p* < 0.05 vs. d, e; d *p* < 0.05 vs. e. In (**E**): a–e *p* < 0.05 vs. control; a *p* < 0.05 vs. b–e; b *p* < 0.05 vs. c; c *p* < 0.05 vs. d, e; d *p* < 0.05 vs. e.

Finally, the role of Schwann cells was also investigated by analyzing the modulation of neuropathic pain in vitro. As reported in Figure 5, after nerve injury induced by 200 ng/mL GGF, myelinating cells were subjected to degradation as demonstrated by p75 analysis and, consequently, to the inhibition of MPZ activity (about 18% and −10%, respectively, *p* < 0.05). At the same time, the PNI condition influenced the expression of neuregulin 1 (NRG1) and the epidermal receptor beta (ERb) activity (*p* < 0.05 compared to control). Conversely, all PEA forms reduced the damage, confirming their positive role in counteracting the demyelination process (all markers had *p* < 0.05 compared to the damage). In particular, the combination of PEA 80mesh and *Equisetum A.*L. induced the greatest effects compared to PEA80mesh alone and PEA-um (*p* < 0.05). Indeed, PEA 80mesh combined with *Equisetum A.*L. was able to reverse the damage by modulating the markers involved in maintaining the myelin sheath to normal activity. In particular, it improved the activity of p75 (Figure 5A) by about 64% compared to PEA 80mesh alone and PEA-um (143%, *p* < 0.05), and MPZ (Figure 5B) by about 50% compared to PEA 80 mesh alone, and this effect was more evident compared to PEA-um (*p* < 0.05). At the same time, NRG1 (Figure 5C) and Erb3 (Figure 5D) activities were increased by the presence of the combination of PEA 80mesh with *Equisetum A.*L. compared to PEA 80mesh alone (about 70% and about 63%, respectively), supporting the hypothesizing of the improvement of both these markers to ameliorate the nerve injury and restore the myelinization process. In this context, the increase in NGF (Figure 5E) due to PEA 80mesh plus *Equisetum A.*L. compared to PEA 80mesh alone (about 23%) and PEA-um (about 100%) confirmed this important mechanism of action.

All these findings suggest that the new combination correctly modulates the biological activity of Schwann cells, also during PNI. Indeed, all these results demonstrated the considerable effect of PEA 80mesh combined with *Equisetum A.*L. in modulating Schwann cells during the development and the relief of nerve injury, suggesting an increased beneficial effect compared to treatments already on the market.

## 3. Discussion

Neuropathic pain is caused by a nerve injury that transfers nociceptive information between the brain and spinal cord and from the skin, muscles, or other body parts. The pain sensation can originate at various levels of the nervous system: the peripheral nerves (Aβ, Aδ and C fibers), the spinal cord, and the brain. Neuropathic pain symptoms can also be very intense and a serious obstacle to leading a normal life. NP remains a problematic disease due to the refractoriness of treatments. Despite advances in neuropathic pain therapy, the effectiveness of treatments remains unsatisfactory to date [38]. NP occurs due to damage and dysfunction of the pain transmission chain (including the peripheral and central nervous systems) [39] and is a common disturbance in the daily lives of many patients. Numerous pieces of evidence have highlighted the role of oxidative stress in neuropathic pain via peripheral and central sensitization, with excessive production of reactive oxygen and nitrogen species (ROS and RNS) causing neuronal damage by interfering with proteins, carbohydrates, lipids, and nucleic acids [3,5]. Over recent years, scientific evidence has been obtained on the connection between dietary regimens or nutrients and the development and progression of different pathologies, including NP, due to the advent of herbal dietary supplements [3], indicating that the future of NP treatment depends on exploring the possibility of combined therapy between pharmacological and non-pharmacological compounds [40]. The current study aimed to identify a powerful food supplement for pain in patients suffering from peripheral nerve injury (PNI) and sensitization using an experimental in vitro model. For this purpose, a combination of nutraceuticals with anti-inflammatory and antioxidant properties was used. Among these, 0.2 µM PEA 80 mesh and 50 µg/ml *Equisetum A.*L. (titrated 10% silica), alone and combined in a new food supplement called EquiPEA™, were used to evaluate the ability to cross the intestinal barrier and reach the peripheral nervous system in order to relieve pain induced by PNI. Transferring our hypothesis into practice, we studied the biological mechanism of action of the new formulation in an in vitro experimental model that reproduces oral administration. In detail, our results showed that the new nutraceutical is able to modulate the main mechanisms underlying nerve fiber damage and, consequently, pain involving the endocannabinoid system. Indeed, as reported in the literature, CB receptors act on GABAergic neurons by entering the physiological mechanism of pain regulation [41]. Accordingly, results revealed that EquiPEA™ acts on the GABAergic system through the CB1 receptor. Furthermore, several studies affirmed that CB receptors on GABAergic neurons might also be needed for modulation of the nervous recovery mechanism located on Schwann cells, which detect nerve injury and provide the first response, playing a critical role in the development and maintenance of neuropathic pain [41]. According to the literature [42,43,44], endogenous cannabinoid ligands, such as PEA [45], and CB1 receptors are widely present in the nociceptive and descending inhibitory pathways. The CB1 receptor is expressed in both nerve endings of GABAergic and glutamatergic neurons, which provides histological evidence that the activation of the CB1 receptor may regulate GABAergic and glutamatergic neurotransmission [43]. In this context, based on the results obtained, it may be possible to hypothesize that EquiPEA™ acts as a CB1R agonist to activate GABAergic and glutamatergic neurons, exerting effects via the CB1 receptor. Therefore, the activation of GABAergic neurons may be necessary for glutamatergic neuron excitation via CB1 receptors to reduce pain. Furthermore, by mimicking in vitro the neuropathy condition, in particular about inflammation and oxidative stress leading to an imbalance of ion transport, data revealed that the combination of PEA 80mesh with *Equisetum A.*L. seems to be able to repair the damage on the myelin sheath that protects the axon and simultaneously be able to act on NGF release and bind the p75 neurotrophin receptor, reproducing the mechanism of analgesia observed in humans. Indeed, as reported above [46,47,48], an increase in NGF levels in various inflammatory conditions may be considered an important hallmark of the human chronic pain condition. Therefore, a reduction in the inflammatory response was important to counteract the negative consequences of chronic pain. However, it is known that when pain occurs, it is accompanied by a functional imbalance between ERb and NRG1, whose relative balance is critical for maintaining Schwann cell homeostasis during PNI. Normally, in peripheral nerves, ERb receptors are activated by NRG1 to regulate growth, migration, differentiation, and dedifferentiation of Schwann cells; alternatively, PNI alters NRG1/ERb signaling by disrupting the balance between NRG1 isoforms, decreasing the expression of several molecules involved in cell survival, and consequently activating the MAPK pathway [49]. While the degeneration of sensory neurons in PNI is clearly associated with impaired neurotrophic support and disruption of NRG-1/ERb B2 signaling, presumably in the biological activity of Schwann cells, treatment with EquiPEA™ restored the altered neurotropism, preventing slowing of nerve conduction and damage to motor fibers. It is well known that the cells respond to the nerve damage by changing their phenotype, proliferating, and interacting with nociceptive neurons by releasing glial mediators (growth factors, cytokines, chemokines, and biologically active small molecules). Furthermore, receptors expressed in active Schwann cells have the potential to regulate their communication with axons, thereby regenerating the myelin sheath and protecting the nerve from further damage.

All these findings support the possible use of a new combination named EquiPEA™ that is able to cross the biological membranes, particularly the intestinal barrier, and reach the target site. Indeed, the effects of EquiPEA™ were analyzed by mimicking human oral administration in vitro. Therefore, the results obtained from the experiments with the 3D model clearly demonstrated the novelty of this new form of PEA, as its bioavailability, absorption, and biodistribution have been improved, allowing effective delivery of the active ingredient to the PNS, where nerve damage is present. This confirms the hypothesis that EquiPEA™ has an excellent absorption profile during intestinal digestion without damaging the intestinal epithelium and maintaining correct intestinal activity.

## 4. Materials and Methods

### 4.1. Agents Preparation

A 0.2 µM PEA 80mesh (donated by Vivatis Pharma Italia srl) and 50 µg/mL *Equisetum A.*L. (titled 10% silica, donated by Vivatis Pharma Italia srl) alone and combined (named EquiPEA™) (Patent Nos. 102022000008066 and 102022000016404 from Futura srl) were used to verify their effectiveness to cross the intestinal barrier and reach the peripheral nervous system. In addition, to analyze the effects of this new combination, the results were compared with other PEA forms present on the market, which have different particle sizes ranging from 177 µm (named PEA80mesh) to 700 µm (called PEAm) (Supplementary Figure S1). These preliminary screenings were used to investigate differences in the PEA market forms in order to use the better PEA market form as a positive control. In particular, ultra-micronized PEA was used as a reference for concentration and mechanism of action. Both the concentrations of PEA and *Equisetum A.*L. were derived from the literature [23,50] and confirmed by dose-response studies that used concentrations ranging from 0.1 µM to 0.4 µM and 25 µg/mL to 100 µg/mL, respectively. All substances tested were prepared directly in Dulbecco’s Modified Eagle’s Medium (DMEM, Merck Life Science, Rome, Italy) without phenol red and supplemented with 0.5% fetal bovine serum (FBS; Merck Life Science, Rome, Italy), 2 mM L-glutamine (Merck Life Science, Rome, Italy), and 1% penicillin–streptomycin (Merck Life Science, Rome, Italy) for all analyses.

### 4.2. Cell Culture

The human epithelial intestinal CaCo-2 cell line, purchased from the American Type Culture Collection (ATCC), was used as an experimental model to predict the features of intestinal absorption following oral intake [51,52]. This cell line was cultured in Dulbecco’s Modified Eagle’s Medium/Nutrient F-12 Ham (DMEM-F12, Merck Life Science, Rome, Italy) containing 10% FBS (Merck Life Science, Rome, Italy), 2 mM L-glutamine, and 1% penicillin–streptomycin (Merck Life Science, Rome, Italy) and maintained in an incubator at 37 °C and 5% CO_2_ [53]. Experiments were carried out using cells at passage numbers between 26 and 32 to maintain the correct paracellular permeability and transport properties [54]. Cells were plated in a variety of ways, including 1 × 10^4^ cells on 96 well plates to study cell viability and ROS production synchronizing cells for 8 h with DMEM without red phenol and supplemented with 0.5% FBS, 2 mM L-glutamine, and 1% penicillin–streptomycin at 37 °C; 2 × 10^4^ cells on 6.5 mm Transwell^®^ (Corning^®^ Costar^®^, Merck Life Science, Rome, Italy) with a 0.4 μm pore polycarbonate membrane insert (Corning^®^ Costar^®^, Merck Life Science, Rome, Italy) in a 24 well plate to perform the absorption analyses [55].

Rat-derived Schwann, RSC-96 cell line, was obtained from ATCC and was cultured in Dulbecco’s Modified Eagle’s Medium (Merck Life Science, Rome, Italy) supplemented with 10% FBS, 2 mM L-glutamine, and 1% penicillin–streptomycin [56], maintaining the cultures at 37 °C with 5% CO_2_. RSC96 cells were subcultured 2–3 times a week, and passages between 10 and 15 were used for the experiments [57].

The rat neuronal PC12 cell line purchased from ATCC was cultured in Roswell Park Memorial Institute-1640 (RPMI, Merck Life Science, Rome, Italy) supplemented with 2 mM glutamine, 10% horse serum (HS; Merck Life Science, Rome, Italy), and 5% FBS. The cultures were maintained at sub-confluency at 37 °C with 5% CO_2_, and the cells used for experiments were between passages 3 and 13 [58]. PC12 cells are one of the most frequently employed neuronal cell lines for in vitro screening for neuroprotective compounds [59]. 4 × 10^6^ RSC96 cells and 1 × 10^5^ PC12 cells were seeded in a co-culture system to reproduce 3D EngNT in vitro in the peripheral nerve environment [56].

### 4.3. Experimental Protocol

The experiments were divided into two steps: the first one investigated the effects of all PEA forms and *Equisetum A*.L. on CaCo-2 cells, analyzing cell viability and ROS production in a dose-response study, and the better concentrations of all substances alone and combined were used in an in vitro intestinal permeability assay to verify the ability to cross the intestinal barrier and maintain a correct TJ activity [60]. In particular, cells were plated in the Transwell^®^ system to verify intestinal integrity by TEER measurement following the treatments. In addition, this in vitro intestinal model was also used to analyze TJ activity by ELISA and the permeability rate by Papp assay. All these experiments were time-dependent from 2 h to 6 h [60]. Moreover, at the end of each stimulation, the basolateral environment was collected to be used to stimulate the 3D EngNT co-culture. In the second step, the 3D EngNT co-culture was used to investigate the effects of the stimulations on the nerve tissue model in vitro after 14 days of culture, maturation time and 24 h of treatments. In this model, mitochondrial metabolism and ROS production were investigated. Finally, additional experiments were also carried out after pretreating for 14 days the 3D EngNT co-culture with 200 ng/mL glial GGF, which is a well-established model to mimic peripheral nerve injuries since it can reproduce robust demyelination [61]. Under demyelination conditions, the 3D EngNT co-culture was stimulated with PEA alone and combined with *Equisetum A.*L. and the ultra-micronized PEA in a commercial form (named PEA from the market), in order to evaluate if this new combination can restore neurite damage. Therefore, mitochondrial metabolism, ROS production, and inflammatory factors were measured; in addition, the re-myelination mechanisms and peripheral nerve recovery were investigated in these conditions. Finally, 10 µM AM251 (Cayman Chemical, Ann Arbor, MI, USA) and 10 µM AM630 (Cayman Chemical, AnnArbor, MI, USA) (CB1 and Cb2 blockers, respectively) [62] were also tested to verify the mechanism of analgesic effect due to the involvement of GABA signaling.

### 4.4. In Vitro Intestinal Barrier Model

In order to evaluate whether all PEA forms and *Equisetum A.*L. samples could be able to cross the intestinal barrier, an in vitro intestinal barrier model was created using the Transwell^®^ system, following a standard protocol reported in the literature [63,64] and approved by the European Medicines Agency (EMA) and Food and Drug Administration (FDA) to predict absorption, metabolism, and bioavailability of several substances after oral intake in humans [65,66]. Briefly, CaCo-2 cells, plated as previously described, were maintained in a complete medium, changing it every other day on the basolateral and apical sides for 21 days before the simulations [63]. During all the maturation time, the TEER values were evaluated by EVOM3, coupled with STX2 chopstick electrodes (World Precision Instruments, Sarasota, FL, USA), to evaluate mature intestinal epithelial formation and a correct paracellular mechanism. On the 21st day, when TEER values were ≥ 400 Ωcm^2^ [67], absorption analysis had started. Before the stimulation, on the apical side, the medium was brought to pH 6.5, the pH in the lumen of the small intestine, while pH 7.4 on the basolateral side represented blood [54,68]. The cells were stimulated with all substances from 2 h to 6 h before the successive analyses, including the permeability assay measured by Papp (cm/s) analysis [53], which follows the following formula:

Papp = dQ/dt ⇥ 1/m0 ⇥ 1/A ⇥ V Donor(1)

dQ: amount of substance transported (nmol or μg);

dt: incubation time (s);

m0: amount of substrate applied to the donor compartment (nmol or μg);

A: surface area of Transwell^®^ membrane (cm^2^);

VDonor: volume of the donor compartment (cm^3^).

Negative controls without cells were tested to exclude the Transwell^®^ membrane’s influence. The analysis was performed in triplicates and reproduced five times.

### 4.5. D EngNT Co-Cultures Setup

The 3D nerve tissue model was prepared according to the literature [56]. The interaction between RSC96 and PC12 cell lines is a key feature for mimicking in vitro the peripheral nerve environment, regenerating neurites, and supporting Schwann cells [56,69,70]. Briefly, 1 mL of a solution containing 80% *v*/*v* Type I rat tail collagen (2 mg/mL in 0.6% acetic acid, Thermo Fischer, Milan, Italy), 10% *v*/*v* Minimum Essential Medium (MEM, Merck Life Science, Milano, Italy), 5.8% *v*/*v* neutralizing solution (Biosystems, Monza, Italy), and 4.2% Schwann cell suspension (4 × 10^6^ RSC96 cells per 1 mL gel) was added to a rectangular scaffold with dimensions of 16.4 mm × 6.5 mm × 5 mm. When the gel had set, it was immersed in 10 mL DMEM and incubated at 37 °C with 5% CO_2_ for 24 h to permit cellular self-alignment; at the end of the time, the gel was stabilized using plastic compression (120 g weight for 1 min). Once the gel had been aligned and stabilized, it was cut into equal segments according to the samples to be treated. Each gel segment was transferred to a 24-well plate, and 1 × 10^5^ PC12 was seeded on top of each segment for establishing the co-cultures; this passage is crucial to permit neurite extension across the horizontal plane following the aligned Schwann gels. The 24-well plate containing gels was incubated for 1 h at 37 °C to allow attachment of neuronal cells to the collagen gel, and then 1 mL of culture medium (containing DMEM supplemented with 10% FBS, 100 U/mL of Penicillin, and 100 μg/mL of Streptomycin, all purchased from Merck Life Science, Milano, Italy) was added to each well.

### 4.6. Cell Viability

The cell viability based on the In Vitro Toxicology Assay Kit (Merck Life Science, Rome, Italy) was verified at the end of each stimulation, following a classical protocol reported in the literature [63]. The absorbance of all solubilized samples (treated and untreated) at 570 nm with correction at 690 nm measured by a spectrometer (Infinite 200 Pro MPlex, Tecan, Männedorf, Switzerland) was expressed by comparing the data to the control sample (untreated samples defined as the 0% line) and reported as the means of five independent experiments performed in triplicate.

### 4.7. ROS Production

The ROS production was quantified by analyzing the reduction of cytochrome C using a standard protocol [53] and measuring the absorbance at 550 nm through the spectrometer (Infinite 200 Pro MPlex, Tecan, Männedorf, Switzerland). O_2_ ratio was expressed as the mean ± SD (%) of nanomoles per reduced cytochrome C per microgram of protein compared to the control (untreated samples) of five independent experiments performed in triplicate.

### 4.8. Occludin Quantification Assay

The human occludin (OCLN) ELISA kit (MyBiosource, San Diego, CA, USA) was used according to the manufacturer’s instructions [53]. CaCo-2 cells were lysed with cold Phosphate-Buffered Saline (PBS, Merck Life Science, Rome, Italy) 1×, centrifuged at 1500× *g* for 10 min at 4 °C, and 100 μL of each sample were analyzed. The enzymatic reaction was measured by a spectrometer (Infinite 200 Pro MPlex, Tecan, Männedorf, Switzerland) at 450 nm. The results were obtained by comparing the data to the standard curve (which ranges from 0 to 1500 pg/mL) and were expressed as a percentage (%) versus the control (0 line) of five independent experiments performed in triplicate.

### 4.9. Claudin-1 ELISA Kit

The human claudin-1 was measured in CaCo-2 lysates by an ELISA kit (Cusabio Technology LLC, Huston, Houston, TX, USA), following the manufacturer’s instructions [53]. CaCo-2 cells were lysed with cold PBS (Merck Life Science, Rome, Italy) 1×, centrifuged at 1500× *g* for 10 min at 4 °C, and 100 μL of each sample were analyzed and read at 450 nm by a spectrometer (Infinite 200 Pro MPlex, Tecan, Männedorf, Switzerland). The results were obtained by comparing the data to the standard curve (which ranges from 0 to 1000 pg/mL) and were expressed as a mean ± SD (%) versus the control (0 line) of five independent experiments performed in triplicate.

### 4.10. Human Tight Junction Protein 1 (ZO-1) Analysis

The human tight junction protein 1 (TJP1) ELISA kit (MyBiosource, San Diego, CA, USA) was used following the manufacturer’s instructions [53]. CaCo-2 cells were lysed with cold PBS (Merck Life Science, Rome, Italy) 1×, centrifuged at 5000× *g* for 5 min at 4 °C, and 100 μL of each sample were analyzed. The plates were read by a spectrometer (Infinite 200 Pro MPlex, Tecan, Männedorf, Switzerland) at 450 nm. The data were obtained and compared to the standard curve (ranging from 0 to 1000 pg/mL), and the results were expressed as a mean ± SD (%) versus the control (0 line) of five independent experiments performed in triplicate.

### 4.11. TNFα Assay

TNFα quantification was obtained using the TNF-α ELISA kit (Merck Life Science, Milano, Italy) according to the manufacturer’s instructions [71]. The absorbance of the samples was measured at 450 nm using a plate reader (Infinite 200 Pro MPlex, Tecan, Männedorf, Switzerland), and the results were expressed as a mean ± SD (%) versus the control (0 line) of five independent experiments performed in triplicate.

### 4.12. Interleukin-2 Assay

Interleukin-2 quantification was determined using the Rat IL-2 (Interleukin-2) ELISA Kit (FineTest, Wuhan, China) according to the manufacturer’s instructions on cell lysates [72]. Briefly, 100 µL of each sample was added into each well, and the plate was incubated at 37 °C for 90 min. At the end of the incubation time, the material in each well was removed, and the wells were washed twice with Wash Buffer. 100 µL of biotin-labeled antibody working solution was added into the above wells, and the plate was incubated at 37 °C for 60 min. At the end of incubation, the solution in each well was removed, and the wells were washed three times with Wash Buffer. Then, 100 µL of SABC Working Solution was added into each well, and the plate was incubated at 37 °C for 30 min. At the end, the wells were washed five times, and 90 µL of TMB substrate were put in each well. After 10–20 min, 50 µL of Stop Solution was put in each well, and the plate was read immediately at 450 nm using a plate reader (Infinite 200 Pro MPlex, Tecan, Männedorf, Switzerland). A standard curve was plotted relating the intensity of the color (OD) to the concentration of standards (ranging from 31.25 to 2000 pg/mL), and the results were expressed as mean ± SD (%) versus control (0 line) of five independent experiments performed in triplicate.

### 4.13. NRG1 Assay

The NRG1 Rat ELISA Kit (FineTest, Wuhan), according to the manufacturer’s instructions, was used in cell culture supernatants. Briefly, 100 µL of each sample was added into each well, and the plate was incubated at 37 °C for 90 min. At the end of the incubation time, the material in each well was removed, and the wells were washed twice with Wash Buffer. 100 µL of biotin-labeled antibody working solution was added into the above wells, and the plate was incubated at 37 °C for 60 min. At the end of incubation, the solution in each well was removed, and the wells were washed three times with Wash Buffer. Then, 100 µL of SABC Working Solution was added into each well, and the plate was incubated at 37 °C for 30 min. At the end, the wells were washed five times, and 90 µL of TMB substrate was put in each well. After 10–20 min, 50 µL of Stop Solution was put in each well, and the plate was read immediately at 450 nm using a plate reader (Infinite 200 Pro MPlex, Tecan, Männedorf, Switzerland). The data were obtained and compared to the standard curve (ranging from 0.156 to 10 ng/mL), and the results were expressed as mean ± SD (%) versus control (0 line) of five independent experiments performed in triplicate.

### 4.14. Myelin Protein Zero Assay

Myelin protein zero (MPZ) production was determined using a Rat ELISA kit (MyBiosource, San Diego, CA, USA) in cell lysates, according to the manufacturer’s instructions. Briefly, 100 µL of each sample was added into each well, and the plate was incubated at 37 °C for 2 h. At the end of the incubation time, 100 µL of biotin antibody was added into the wells, and the plate was incubated at 37 °C for 60 min. At the end of incubation, the solution in each well was removed, and the wells were washed three times with Wash Buffer. Then, 100 µL of HRP-avidin solution was added into each well, and the plate was incubated at 37 °C for 1 h. At the end, the wells were washed five times, and 90 µL of TMB substrate was put in each well. After 30 min at 37 °C, 50 µL of Stop Solution was put in each well, and the plate was read immediately at 450 nm using a spectrometer (Infinite 200 Pro MPlex, Tecan, Männedorf, Switzerland). The concentration was expressed as ng/mL compared to a standard curve (ranging from 0.06 to 18 ng/mL), and the results were reported as the mean ± SD (%) versus the control (0 line) of five independent experiments performed in triplicate.

### 4.15. NGFR Assay

The Rat NGFR ELISA kit (Thermo Fischer, Milan, Italy) (MyBiosource, San Diego, CA, USA), according to the manufacturer’s instructions, was used on cell lysates. Briefly, 100 µL of each sample was added into each well, and the plate was incubated at 37 °C for 2 h. At the end of the incubation time, 100 µL of biotin antibody was added into the wells, and the plate was incubated at 37 °C for 60 min. At the end of incubation, the solution in each well was removed, and the wells were washed three times with Wash Buffer. Then, 100 µL of HRP-avidin solution was added into each well, and the plate was incubated at 37 °C for 1 h. At the end, the wells were washed five times, and 90 µL of TMB substrate was put in each well. After 30 min at 37 °C, 50 µL of Stop Solution was put in each well, and the plate was read immediately at 450 nm using a spectrometer (Infinite 200 Pro MPlex, Tecan). The data were obtained and compared to the standard curve (ranging from 0.312 to 20 ng/mL), and the results were expressed as a mean ± SD (%) versus control (0 line) of five independent experiments performed in triplicate.

### 4.16. Human Beta-NGF Assay

The Rat beta-NGF ELISA kit (Abcam, Cambridge, UK) was used in cell lysates following the manufacturer’s instructions [73]. Briefly, 100 μL of diluted samples were incubated overnight at 4 °C, washed four times with 1× Wash Buffer, and then 100 μL of detection antibody was added to each well and incubated for 1 h at room temperature with gentle shaking. Then the wells were washed four times, and 100 μL of streptavidin-HRP and the plate were incubated for 45 min. After the incubation, the wells were washed again, and 100 μL of TMB substrate were added. Finally, the plate was incubated for 30 min at room temperature in the dark with gentle shaking, and the reaction was stopped with 50 μL of Stop Solution. The absorbance was measured by the spectrometer at 450 nm (Infinite 200 Pro MPlex, Tecan, Männedorf, Switzerland), and expressed as pg/mL compared to a standard curve (ranging from 15 to 15,000 pg/mL), and the results were reported as the mean ± SD (%) versus control of five independent experiments performed in triplicate.

### 4.17. Estrogen Receptor Beta Assay

The Rat Estrogen Receptor Beta (ERb) ELISA Kit (Cloud-Clone, Houston, TX, USA) was used on cell lysates, according to the manufacturer’s instructions [74]. Briefly, 100 µL of each sample was added into each well, and the plate was incubated at 37 °C for 1 h. At the end of all reactions, 50 µL of Stop Solution was put in each well, and the plate was read immediately at 450 nm using a spectrometer (Infinite 200 Pro MPlex, Tecan). The concentration was obtained and compared to a standard curve (ranging from 0.312 to 20 ng/mL), and the results were expressed as the mean ± SD (%) versus control of five independent experiments performed in triplicate.

### 4.18. Gamma-Aminobutyric Acid Assay

The Rat GABA (Gamma-aminobutyric acid) ELISA Kit (FineTest, Wuhan, China) on cell lysates was performed following the manufacturer’s instructions [75]. Briefly, 50 μL of the sample was added with 50 μL of biotin-labeled antibody in each well and incubated for 45 min at 37 °C; then the plate was washed 3 times, 100 μL of SABC working solution was added in each well, and the plate was incubated for 30 min at 37 °C. At the end of incubation, each well was washed five times, and then 90 μL of TMB substrate solution was added to the wells before incubating the plate at 37 °C for 15 min. Then 50 μL of stop solution was added to each well, and the absorbance was measured by the spectrometer at 450 nm (Infinite 200 Pro MPlex, Tecan, Männedorf, Switzerland). The concentration was analyzed and compared to a standard curve (ranging from 6 to 400 pg/mL), and the results generated were expressed as the mean ± SD (%) versus control of five independent experiments performed in triplicate.

### 4.19. Western Blot

3D EngNT co-culture was lysed in ice with Complete Tablet Buffer (Roche, Basilea, Svizzera) supplemented with 2 mM sodium orthovanadate (Na_3_VO_4_), 1 mM phenylmethanesulfonyl fluoride (PMSF) (Merck Life Science, Rome, Italy), a 1:50 mix of Phosphatase Inhibitor Cocktail (Merck Life Science, Rome, Italy), and a 1:200 mix of Protease Inhibitor Cocktail (Merck Life Science, Rome, Italy). According to the standard protocol, 30 μg of protein from each sample was resolved on 8% or 10% SDS-PAGE gels, and polyvinylidene difluoride membranes (PVDF, GE, Healthcare Europe GmbH) were incubated overnight at 4 °C with the following specific primary antibodies: anti-CB1 (1:500; Santa Cruz, CA, USA), and anti-CB2 (1:500; Santa Cruz, CA, USA). Protein expression was normalized and verified through anti-β-actin detection (Merck Life Science, Rome, Italy). The results were expressed as means ± SD (% vs. control).

### 4.20. Statistical Analysis

Data collected were processed using Prism GraphPad statistical software 9.4.1 using one-way analysis of variance (ANOVA), followed by Bonferroni post hoc tests. Comparisons between the two groups were performed using a two-tailed Student’s *t*-test. Multiple comparisons among groups were analyzed by a two-way ANOVA followed by a two-sided Dunnett post hoc test. All results were expressed as the mean ± SD of at least 5 independent experiments produced in triplicates. Differences with a *p* < 0.05 were considered statistically significant.

## 5. Conclusions

Conventional analgesic therapies, such as non-steroidal anti-inflammatory drugs, exhibit several adverse side effects that may alter the positive outcome of treating neuropathies. Hence, the search for alternative therapeutics to attenuate peripheral neuropathic pain has led researchers to identify innovative drugs from natural sources. Therefore, we have found a viable alternative to the common analgesic drugs for attenuating the pain caused by damage to the peripheral nerves. Indeed, our results support the hypothesis that *Equisetum A.*L. and PEA effectively attenuate neuropathy, modulate the pain key mechanism, and activate the recovery mechanisms of PNS cells. In conclusion, the present work provides important insights into the effects of nutraceuticals in alleviating neuropathic pain conditions and how EquiPEA™ can be considered an innovative and safe remedy for neuropathies.

## Figures and Tables

**Table 1:** Analysis of cell viability measured by the MTT test on CaCo-2 cells. The effects of and *Equisetum A.*L., both alone and combined, were compared to PEA-um at 3 and 6 h. *Equisetum* A.L. = *Equisetum arvense* L.; PEA-um = PEA ultra-micronized. The data are the mean ± SD (%) of five independent experiments performed in triplicate and normalized to control values (0%).

		PEA-um			PEA 80mesh			*Equisetum A.*L.			PEA 80mesh + *Equisetum A.*L.
	Dose	0.1 µM	0.2 µM	0.4 µM	0.1 µM	0.2 µM	0.4 µM	25 µg/mL	50 µg/mL	100 µg/mL	0.2 µM + 50 µg/mL
Time											
**3 h**		80 ± 1.71	90 ± 1.21	72 ± 1.46	85 ± 0.83	92 ± 1.23	70 ± 1.48	45 ± 0.85	49 ± 1.48	46 ± 1.57	94 ± 1.33
**6 h**		76 ± 1.48	87 ± 1.67	70 ± 1.11	78 ± 1.74	90 ± 1.26	68 ± 1.27	30 ± 1.27	44 ± 1.22	40 ± 1.57	91 ± 1.07

**Table 2:** Analysis of ROS production measured by cytochrome C reduction on CaCo-2 cells. The effects of PEA80mesh and *Equisetum A.*L., alone and combined, compared to PEA-um at 3 h and 6 h, were analyzed. *Equisetum A*.L. = *Equisetum arvense* L.; PEA-um = PEA ultra-micronized. The data are the mean ± SD (%) of five independent experiments performed in triplicate and normalized to control values (0%).

		PEA-um			PEA 80mesh			*Equisetum A.*L.			PEA 80mesh + *Equisetum A.*L.
	Dose	0.1 µM	0.2 µM	0.4 µM	0.1 µM	0.2 µM	0.4 µM	25 µg/mL	50 µg/mL	100 µg/mL	0.2 µM + 50 µg/mL
Time											
**3 h**		4 ± 1.14	5 ± 1.67	7 ± 1.48	3 ± 1.78	2 ± 1.74	1 ± 1.19	−8 ± 1.74	−9 ± 1.46	−9 ± 1.71	−15 ± 0.89
**6 h**		8 ± 1.15	7 ± 1.56	10 ± 1.22	7 ± 1.41	6 ± 1.38	5 ± 1.47	−9 ± 1.38	−9 ± 1.17	−9 ± 1.48	−12 ± 1.23

## Data Availability

Raw data are preferably deposited at the Laboratory of Physiology (C. Molinari), ensuring appropriate measures so that raw data are retained in full forever under a secure system. The data presented in this study are available upon reasonable request from the corresponding author.

## Supplementary Materials

The following supporting information can be downloaded at: https://www.mdpi.com/article/10.3390/ijms24065503/s1.

## Abbreviations

3D EngNT	3D Engineered neural tissue
ATCC	American Type Culture Collection
BDNF	Brain-derived neurotrophic factor
CB1R	cannabinoid receptor 1
CB2R	cannabinoid receptor 2
COX-2	Cyclooxygenase-2
DMEM	Dulbecco’s modified Eagle’s medium
ELISA	Enzyme-Linked Immunosorbent Assay
EMA	European Medicines Agency
ERb	Estrogen Receptor beta
*Equisetum A*.L.	*Equisetum arvense* L.
FAAH	Fatty acid amide hydrolase
FBS	foetal bovine serum
FDA	US Food and Drug Administration
GABA	gamma-amino butyric acid
GAD	Glutamic acid decarboxylase
GGF	glial growth factor 2
IASP	International Association for the Study of Pain
IL-1β	Interleukin-1 beta
IL-2	I nterleukin-2
MEM	Minimum Essential Medium
MPZ	Myelin protein zero
MTT	3-(4,5-Dimethylthiazol-2-yl)-2,5-diphenyltetrazolium bromide
NGF	Nerve growth factor
NP	neuropathic pain
NRG1	Neuregulin 1
OS	oxidative stress
Papp	apparent permeability coefficient
PBS	phosphate buffered saline
PEA	palmitoylethanolamide
PEA-um	ultra-micronized PEA
PEAm	micronized PEA
PNI	peripheral nervous injury
PNS	peripheral nervous system
PVDF	polyvinylidene difluoride
RNS	Radical nitrogen species
ROS	radical oxygen species
RPMI	Roswell Park Memorial Institute-1640
RSC-96	Rat-derived Schwann
SIRT1	sirtuin 1
SIRT3	sirtuin 3
TEER	transepithelial electrical resistance
TJ	Tigh Junction
TNFα	Tumour Necrosis Factor alpha
ZO-1	Human Tight Junction Protein 1
