# Supplementary material for: Novel Approach to the Treatment of Neuropathic Pain Using a Combination with Palmitoylethanolamide and Equisetum arvense L. in an In Vitro Study
Source: Int J Mol Sci. 2023 Mar 13;24(6):5503. doi: 10.3390/ijms24065503 (PMC10053612; doi:10.3390/ijms24065503)
Supplement: Supplementary file 1 [file ijms-24-05503-s001.zip › ijms-2198861-supplementary.pdf]

International Journal of
Molecular Sciences

MDPI

Supplementary Material

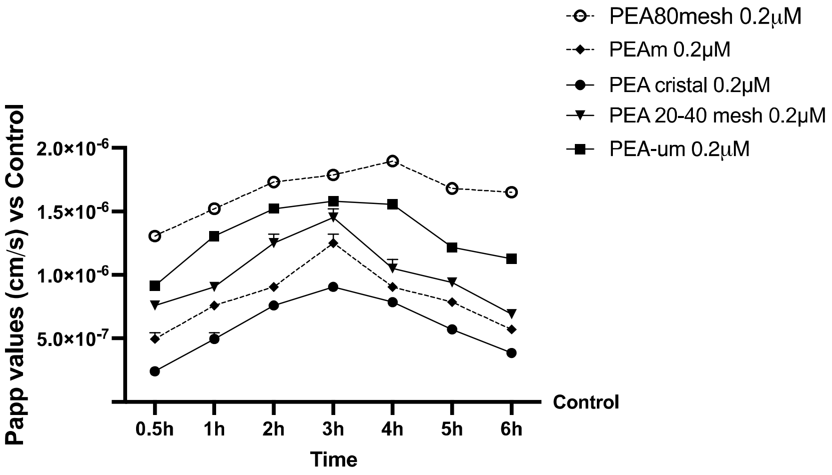

Sample	1h	2h	3h	4h	5h	6h
PEA80mesh 0.2μM	44%	52%	58%	62%	48%	36%
PEAm 0.2μM	22%	28%	31%	35%	33%	25%
PEA cristal 0.2μM	12%	18%	21%	25%	23%	19%
PEA20-40mesh 0.2μM	30%	33%	38%	43%	35%	28%
PEAum 0.2μM	40%	47%	50%	53%	45%	32%

Figure S1. Permeability study on CaCo-2 cells. Regarding the Papp values data < 0.2×10^{-6} cm/s means very poor absorption with a bioavailability < 1 %, data between 0.2×10^{-6} and 2×10^{-6} cm/s with bioavailability between 1 and 90 %, and data > 2×10^{-6} cm/s means very good absorption with a bioavailability over 90 %. PEAm=PEA micronized, PEA-um= PEA ultra-micronized.
